# Blood based metabolic markers of glioma from pre-diagnosis to surgery

**DOI:** 10.1038/s41598-024-71375-6

**Published:** 2024-09-05

**Authors:** Sebastian Löding, Henrik Antti, Rickard L. Sjöberg, Beatrice Melin, Benny Björkblom

**Affiliations:** 1 Department of Chemistry, Umeå University, Linnaeus väg 10, 901 87 Umeå, Sweden; 2 Department of Clinical Science, Neurosciences, Umeå University, 901 85 Umeå, Sweden; 3 Department of Diagnostics and Intervention, Oncology, Umeå University, 901 87 Umeå, Sweden

**Keywords:** Glioma, Glioblastoma, Blood metabolites, Early detection, Surgery, Liquid biopsy, Diagnostic markers, CNS cancer

## Abstract

Gliomas are highly complex and metabolically active brain tumors associated with poor prognosis. Recent reports have found altered levels of blood metabolites during early tumor development, suggesting that tumor development could be detected several years before clinical manifestation. In this study, we performed metabolite analyses of blood samples collected from healthy controls and future glioma patients, up to eight years before glioma diagnosis, and on the day of glioma surgery. We discovered that metabolites related to early glioma development were associated with an increased energy turnover, as highlighted by elevated levels of TCA-related metabolites such as fumarate, malate, lactate and pyruvate in pre-diagnostic cases. We also found that metabolites related to glioma progression at surgery were primarily high levels of amino acids and metabolites of amino acid catabolism, with elevated levels of 11 amino acids and two branched-chain alpha-ketoacids, ketoleucine and ketoisoleucine. High amino acid turnover in glioma tumor tissue is currently utilized for PET imaging, diagnosis and delineation of tumor margins. By examining blood-based metabolic progression patterns towards disease onset, we demonstrate that this high amino acid turnover is also detectable in a simple blood sample. These findings provide additional insight of metabolic alterations during glioma development and progression.

## Introduction

Glioma is the most common type of malignant primary brain tumor in adults. The current treatment approaches include surgical resection often combined with radiochemotherapy, yet the median survival time of the most aggressive subtype, glioblastoma, is no longer than 15 months^1^. Given the poor prognosis and limited treatment options, early detection of glioma development is important in order to treat patients at an earlier stage of the disease progression. Interestingly, markers of glioma development have been reported to appear several years before a patient is diagnosed with a brain tumor^2,3^. These markers include altered metabolite levels in blood, which have been found in several studies^2,4–7^. Recently, it was shown that glioma development can be detected in blood up to at least two years before diagnosis with a panel of 20 metabolites in a multicenter pan-European cohort^7^. These findings highlight the possibility of an earlier detection of glioma, which could potentially lead to an improved prognosis and outcome for glioma patients. Moreover, analyzing metabolic alterations related to disease development and progression could enhance our understanding of the mechanism of the disease.

Metabolic changes associated with glioma include elevated levels of certain metabolites related to an increased energy turnover^7^. These include metabolites within the tricarboxylic acid (TCA) cycle and other metabolites associated with an increased energy turnover such as hypoxanthine, lactate, and N-lactoyl-amino acids^8,9^. Other metabolic changes related to glioma development include an imbalanced redox homeostasis and altered glutathione metabolism^2,4,7^. Previous studies have mainly focused the analysis on pre-diagnostic plasma samples or samples collected after glioma diagnosis^10^. To fully understand changes in metabolite levels during the timeline of glioma development and progression, it would be necessary to analyze both pre-diagnostic samples and surgical samples over time from the same individuals.

In this study, we examined blood plasma samples collected years before diagnosis, as well as on the day of surgery. We compared pre-diagnostic plasma samples with matched healthy controls to find early metabolic markers related to glioma development. We also analyzed surgery samples in relation to pre-diagnostic samples, from the same individuals, to find metabolites related to glioma progression. All samples were analyzed by untargeted gas chromatography mass spectrometry (GC–MS) based metabolite analyses. We also performed targeted GC–MS analyses, in an attempt to validate previous findings of elevated levels of N-lactoyl-amino acids close to glioma diagnosis.

## Results

### Altered metabolite levels related to glioma development

**Fig. 1:**
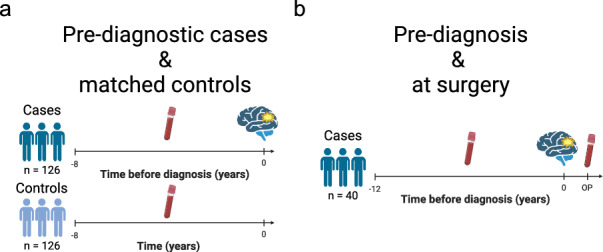
Study design and analysis overview. (**a**) Collection of plasma samples from pre-diagnostic glioma cases (n = 126) within eight years before diagnosis, along with samples from matched healthy controls (n = 126). (**b**) Plasma samples were also collected on the day of glioma surgery (n = 40) from individuals who had previously donated a pre-diagnostic sample within twelve years. The illustration was created with BioRender.com.

**Fig. 2:**
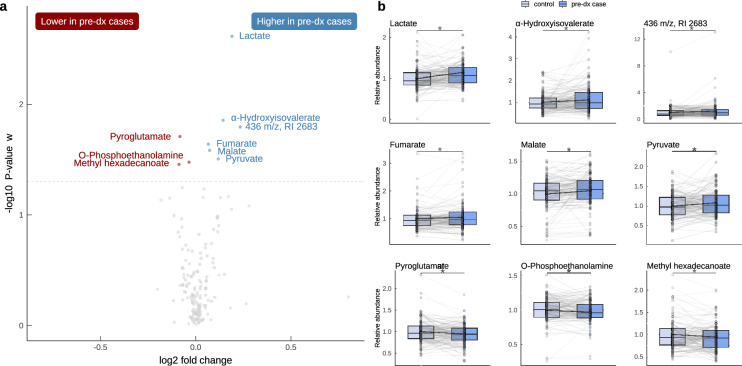
Metabolite levels in plasma from pre-diagnostic glioma cases compared to controls. (**a**) Volcano plot of all 134 metabolites and 22 unidentified molecular features for case–control pairs in the NSHDS discovery cohort within eight years before diagnosis (n = 126 pairs). Effect sizes are shown as log_2_ fold change, and statistical significance by -log_10_ *P*-value for individual metabolites using model loadings w. Metabolites with significantly higher (blue) or lower (red) levels in plasma from pre-diagnostic glioma cases are named. (**b**) Paired-boxplots of the significant metabolites displayed in (a).

To find metabolites that could indicate early glioma development, we analyzed plasma samples from patients that had been collected up to eight years before glioma diagnosis, and carefully matched plasma samples from cancer free healthy controls (Table 1 and Fig. 1). All samples were part of the Northern Sweden Health and Disease Study (NSHDS), which is a population based cohort with over 150,000 individuals^11^. Based on our criteria, we retrieved 126 pre-diagnostic glioma plasma samples together with 126 matched control samples. By use of untargeted GC–MS analysis, we identified 134 metabolites with high confidence, and 22 molecular features with unknown identity. The study design enabled us to do dependent analyses as each pre-diagnostic case was paired to a non-disease control based on stringent matching criteria (see Methods section) and differences in metabolite levels were calculated. Paired multivariate statistical analysis was applied by using Orthogonal Projections to Latent Structures – Effect Projection (OPLS-EP). Initial OPLS-EP modeling did not show a significant difference between pre-diagnostic cases and controls based on all metabolites combined (*P* = 1). However, when we examined the significance level of individual metabolites, eight identified metabolites and one unidentified molecular feature showed significantly altered levels in pre-diagnostic samples (Fig. 2, Table 2). The significant metabolites replicate previous findings of elevated levels of lactate, fumarate, malate and pyruvate in cases within eight years to glioma diagnosis in the NSHDS, analyzed by the liquid chromatography analytical platform at Metabolon Inc^7^. Additionally, we found elevated levels of α-hydroxyisovalerate, and lower levels of pyroglutamate (also known as 5-oxoproline), O-phosphoethanolamine and methyl hexadecanoate in pre-diagnostic glioma cases (Fig. 2, Table 2).

**Table 1:** Characteristics of pre-diagnostic cases and their matched controls, along with samples obtained at the time of surgery.

Patient and sample characteristics		
**Pre-diagnosis**	Cases	Controls
Subjects, *n*	126	126
Sex, *n* (%)		
Female	80 (63.5)	80 (63.5)
Male	46 (36.5)	46 (36.5)
Age at sampling (years), mean (range)	54.3 (28.6–73.6)	54.3 (27.8–73.0)
Time from sampling to diagnosis (years), mean (range)	3.8 (0.15–7.98)	n/a
Sampling date (year), median (range)	1999 (1988–2014)	1999 (1988–2014)
Time in freezer (years), mean (range)	19.3 (5.8–31.3)	19.3 (5.8–31.3)
BMI (kg/m^2^), mean (range)	25.6 (18.3–39.8)	25.5 (18.1–35.0)
Fasting status, *n*		
0–4 h	42	45
4–6 h	11	8
> 8 h	73	73
Glioma subtype, *n* (females/males)		
Glioblastoma: 9440/3	79 (45/34)	n/a
Gliosarcoma: 9442/3	1 (0/1)	n/a
Astrocytoma: 9400/3, 9401/3	24 (19/5)	n/a
Oligodendroglioma: 9450/3, 9451/3	16 (11/5)	n/a
Glioma NOS: 9380/3	6 (5/1)	n/a
**At surgery**	Cases	Cases with < 7 years to pre-diagnostic sample
Subjects, *n*	40	27
Sex, *n* (%)		
Female	17 (42.5)	11 (40.7)
Male	23 (57.5)	16 (59.3)
Age at diagnosis (years), mean (range)	62.0 (43.8–77.3)	62.6 (43.8–77.3)
Glioma subtype, *n* (females/males)		
Glioblastoma: 9440/3	29 (11/18)	21 (8/13)
Gliosarcoma: 9442/3	1 (0/1)	0
Astrocytoma: 9400/3, 9401/3	5 (3/2)	4 (2/2)
Oligodendroglioma: 9450/3, 9451/3	5 (3/2)	2 (1/1)

**Table 2:** Metabolites with significantly higher or lower levels in pre-diagnostic cases within eight years to glioma diagnosis.

Metabolite	*P*-value	Mean difference (%)	HMDB ID
Higher in pre-diagnostic cases			
Lactate	0.002	14	HMDB0000190
α-Hydroxyisovalerate	0.014	10	HMDB0000407
Fumarate	0.023	5	HMDB0000134
Malate	0.026	5	HMDB0000156
Pyruvate	0.031	9	HMDB0000243
Lower in pre-diagnostic cases			
Pyroglutamate	0.020	− 6	HMDB0000267
O-Phosphoethanolamine	0.033	− 2	HMDB0000224
Methyl hexadecanoate	0.035	− 6	HMDB0061859

*P*-values and mean percentage difference were calculated from case–control pairs within eight years to diagnosis (n = 126). Significance levels were calculated from loadings w of the OPLS-EP model (two-sided), which is equivalent to paired samples *t*-test.

Since glioma is a heterogenous disease with several subtypes, we performed the same analysis separately for pre-diagnostic glioblastoma cases (n = 79) and their paired controls (n = 79). Here as well, we did not generate a significant OPLS-EP model (*P* = 1). When we examined the metabolites individually, malate, fumarate, α-hydroxyisovalerate, pyruvate and lactate were significantly elevated in pre-diagnostic glioblastoma patients (Supplementary Table S1), which they also were when all glioma pre-diagnostic cases were analyzed together (Fig. 2, Table 2). It should be noted that the elevated levels of these metabolites were higher in pre-diagnostic glioblastoma cases compared to when all glioma were analyzed together. In addition, pre-diagnostic glioblastoma cases had significantly elevated levels of both α-hydroxybutyrate and β-hydroxybutyrate, together with elevated levels of oxalate, 2,3-dihydroxybutanoate and 1,5-anhydrosorbitol (Supplementary Table S1).

### Altered metabolite levels related to glioma progression

**Fig. 3:**
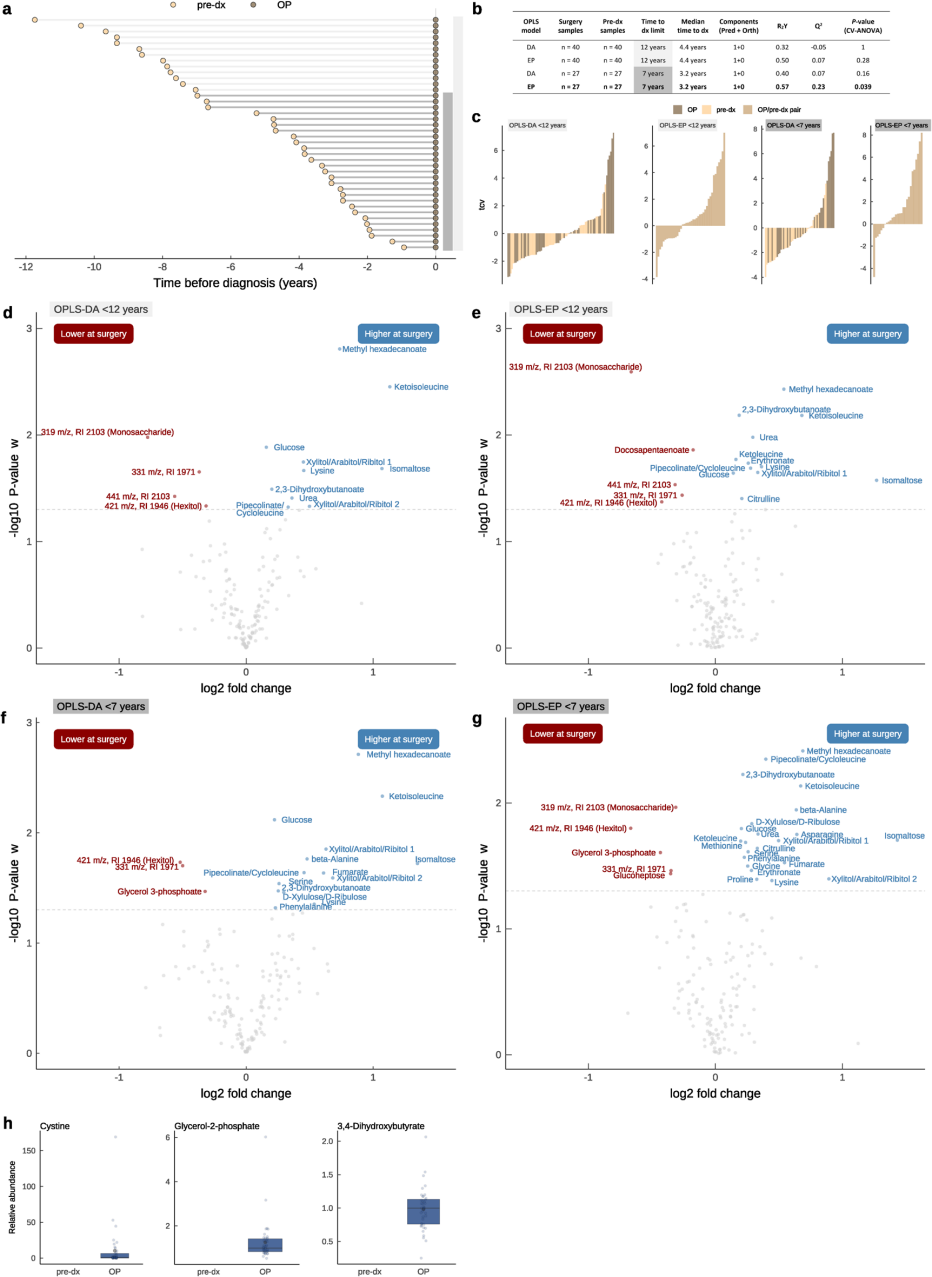
Metabolite levels in plasma on the day of surgery compared to years before diagnosis. (**a**) An overview of repeated plasma samples, collected up to 12 years before glioma diagnosis (pre-dx) and on the day of surgery (OP), from the same individuals (n = 40). (**b**) Summary of the four generated OPLS-models. Bold text highlight significant OPLS-EP model (n = 27 pairs) with satisfactory Q^2^ value and P < 0.05. (**c**) Cross-validated scores of the four OPLS-models shown in (b). (**d**–**g**) Volcano plots of the 134 metabolites and 22 unidentified molecular features for the repeated plasma samples modeled in (**b**). Effect sizes are shown as log_2_ fold change, and statistical significance by -log_10_ *P*-value for individual metabolites using model loadings w. Metabolites with significantly higher (blue) or lower (red) levels in surgery samples are named. (**h**) Boxplots of metabolites that were only detected in surgery samples.

To find metabolites related to glioma progression, we analyzed plasma samples collected on the day of surgery (n = 40) for a subset of individuals that had previously donated a pre-diagnostic plasma sample up to 12 years before surgery. This approach allowed us to examine changes of metabolite levels over time, from years before diagnosis to the time of surgery (Table 1 and Fig. 1) of the 134 identified metabolites detected and unidentified 22 molecular features. Surgery samples were obtained from the Uppsala-Umeå Comprehensive Cancer Consortium (U-CAN)^12^, and pre-diagnostic samples were sourced from NSHDS, as previously described. All 40 individuals were fasting during both sample collections. An overview of time between collection of the surgery and pre-diagnostic samples is shown in Fig. 3a. Before statistical analysis, each surgery sample was paired with its corresponding pre-diagnostic sample, and changes in metabolite levels were calculated. OPLS-EP modeling was applied to find metabolites related to disease progression. To explore how paring affects the quality of models and metabolite discovery, we also performed independent sample analysis by OPLS—Discriminant Analysis (OPLS-DA). We initially generated an OPLS-DA model with all surgery samples (n = 40) and all matched pre-diagnostic samples (n = 40), and an OPLS-EP model with all surgery and pre-diagnostic sample pairs (n = 40 pairs) (Fig. 3b). The OPLS-DA model generated a *P*-value of 1 while the OPLS-EP *P*-value was improved to 0.28. Even though none of the models were significant, it indicates that the paired-sample approach results in higher information retrieval and increased signal to noise ratio. This was also observed when looking at the cross-validated scores of the two models (Fig. 3c), were the paired analysis correctly classified the samples to a greater extent than the unpaired analysis. Upon examining individual metabolites, it was clear that the paired analysis performed better in terms of a larger number of metabolites being significant (Fig. 3d,e).

With the aim of capturing the timeline of glioma development, we limited the time between collection of the pre-diagnostic sample to seven years to diagnosis. We chose this rationale since previous studies have indicated that glioma development might start up to 8 years before diagnosis^2,3,7^. Moreover, it has also been seen that longitudinal plasma metabolite analysis is improved when time between samplings is limited to 7 years^2^. Therefore, we generated an OPLS-DA model and an OPLS-EP model using pre-diagnostic cases sampled within 7 years to diagnosis (n = 27) together with their corresponding surgery sample (n = 27) (Fig. 3b). As hypothesized, the significance of the models improved with *P*-values of 0.16 and 0.039 for the OPLS-DA and OPLS-EP model, respectively. It was also evident that the dependent sample analysis by OPLS-EP analysis performed better compared to the independent sample analysis by OPLS-DA, both in terms of model quality parameter (Fig. 3b) and the extent of correct sample classification (Fig. 3c). The paired analysis also increased the number of significant metabolites compared to the unpaired analysis (Fig. 3c–g), with 22 metabolites significantly higher at the time of surgery and 2 metabolites and 3 unidentified molecular features significantly lower at surgery (Table 3).

**Table 3:** Metabolites with significantly higher or lower levels at surgery.

Metabolite	*P*-value	Mean difference (%)	HMDB ID
Higher at time of surgery			
Methyl hexadecanoate	0.004	61	HMDB0061859
Pipecolinate/Cycloleucine	0.004	32	HMDB0000070/ HMDB0062225
2,3-Dihydroxybutanoate	0.006	16	HMDB0245394
Ketoisoleucin	0.007	59	
beta-Alanine	0.011	55	HMDB0000056
Xylulose/Ribulose	0.015	22	
Glucose	0.016	15	HMDB0000122
Urea	0.018	26	HMDB0000294
Asparagine	0.018	56	HMDB0033780
Isomaltose	0.020	170	HMDB0002923
Xylitol/Arabitol/Ribitol 1	0.020	41	
Ketoleucine	0.020	15	HMDB0000695
Methionine	0.021	18	HMDB0000696
Citrulline	0.023	26	HMDB0000904
Serine	0.024	20	HMDB0000187
Phenylalanine	0.027	17	HMDB0000159
Fumarate	0.030	46	HMDB0000134
Glycine	0.032	19	HMDB0000123
Erythronate	0.034	22	HMDB0000613
Xylitol/Arabitol/Ribitol 2	0.040	86	
Proline	0.040	25	HMDB0251528
Lysine	0.041	36	HMDB0000182
Lower at time of surgery			
Monosacharide (319 m/z, RI 2103)	0.011	-24	
Hexitol (421 m/z, RI 1946)	0.016	-59	
Glycerol 3-phosphoate	0.025	-35	HMDB0000126
Unknown (331 m/z, RI 1971)	0.035	-27	
Glucoheptose	0.036	-28	

*P*-values and mean percentage difference were calculated from paired surgery samples (n = 27) and pre-diagnostic samples (n = 27) within seven years to diagnosis. Significance levels were calculated from loadings w of the OPLS-EP model (two-sided), which is equivalent to paired samples *t*-test.

It was notable that many of the metabolites that were higher at surgery were either amino acids or metabolites of amino acid metabolism (Fig. 3g). Significantly elevated amino acids were asparagine, methionine, citrulline, serine, phenylalanine, glycine, proline, lysine, beta-alanine and pipecolinate/cycloleucine. Metabolites of amino acid metabolism included the branched-chain alpha-ketoacids (BCKAs) namely ketoisoleucine and ketoleucine, which are products of the branched-chain amino acids (BCAAs) isoleucine and leucine catabolism. Notably, the levels of fumarate which was found to be higher in pre-diagnostic cases compared to controls, were even higher at surgery. Moreover, cystine, glycerol-2-phosphate and 3,4-dihydroxybutyrate were only detected in samples collected at the time of surgery (Fig. 3h).

To examine if altered metabolite levels at surgery depends on glioma subtype, we performed OPLS-EP on glioblastoma patients only with a limit of seven years between the surgery sample and the pre-diagnostic sample (n = 21 pairs). The model did not reach statistical significance (*P* = 0.14), which was likely due to loss of power when fewer samples were included. A large overlap of significant metabolites was seen for glioblastoma separately and all glioma (Supplementary Table S2). Interestingly, α-aminobutyrate and α-hydroxyisovalerate was significantly elevated in glioblastoma patients at surgery. As showed earlier, α-hydroxyisovalerate was also significantly elevated in pre-diagnostic glioma cases compared to healthy controls (Fig. 2).

Investigating blood samples from biobanks of human populations to identify systematic differences related to diseases can pose difficulties due to the existence of other sources of systematic variation. We can successfully obtain the pattern of progression by subtracting one sample from the repeated sample, which normalizes individual differences and highlights changes over time. By comparing this progression pattern with a closely matched control, one can further mitigate extraneous influences linked to the passage of time and sample storage^2^. However, in the context of the surgical samples in this study, we lack controls. In order to assess the impact of the time interval between samples as a covariate in our discovery of metabolites, we generated an OPLS model with time between the paired pre-diagnostic and surgery samples as Y variable and differences in metabolites levels as X variables (Supplementary Fig. S1a). Upon examining metabolites, we found that metabolites that are related to time between the samples are in general not the same metabolites we discovered to be related with disease progression (Supplementary Fig. S1b). This was shown as the significantly altered metabolites at surgery were not observed to be the most important coefficients of the time model (Supplementary Fig. S1c).

### Altered metabolite levels years before glioma diagnosis and at surgery

**Fig. 4:**
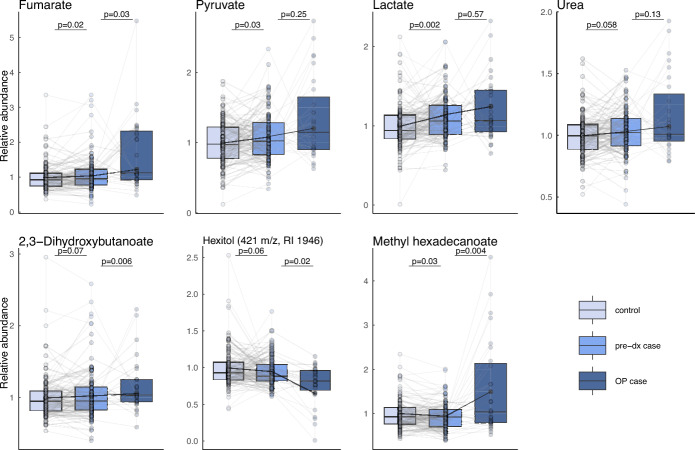
Metabolites that show altered levels in blood years before and at surgery. Paired-boxplots of pre-diagnostic (pre-dx) cases and controls (n = 126 pairs), and paired samples from surgery (OP case) and years before diagnosis (pre-dx) (n = 27 pairs).

As shown, the levels of several metabolites are significantly altered years before glioma diagnosis and at the time of surgery. To bridge the two separate analyses and to find metabolic markers of early diagnostic potential, we targeted the most altered metabolites in pre-diagnostic cases compared to controls (p < 0.10) and analyzed how their levels progressed towards surgery (Fig. 4). As reported, fumarate shows significantly elevated levels in pre-diagnostic cases compared to controls, and its levels are further elevated to the time of surgery. The same pattern of elevated levels years before diagnosis and even higher levels at the time of surgery was also seen for pyruvate, lactate, urea and 2,3-dihydroxybutanoate (Fig. 4). On the other hand, an unidentified hexitol (421 m/z, RI 1946) showed lower level in pre-diagnostic cases compared to controls and even lower levels at the time of surgery (Fig. 4). Lastly, methyl hexadecanoate showed significantly lower levels years before diagnosis and significantly elevated levels at the time of surgery (Fig. 4).

### Targeted analysis of N-lactoyl-amino acids by GC–MS

As observed in this study, amino acids and their metabolism play a role in glioma development and progression, with higher levels at the time of surgery. A class of metabolites that have recently garnered increased interest are N-lactoyl-amino acids^9,13^. Using a LC–MS/MS based approach, we previously detected elevated levels of N-lactoyl-amino acids in blood of pre-diagnostic glioma cases^7^. To validate these findings with another method, we performed targeted GC–MS analysis of four commercially available N-lactoyl-amino acids: N-lactoyl-phenylalanine, N-lactoyl-leucine, N-lactoyl-valine and N-lactoyl-tyrosine (Supplementary Fig. S2). Although our method could detect N-lactoyl-amino acid standards down to 1.3 pmol levels, corresponding N-lactoyl-amino acids within our pre-diagnostic glioma samples were below our methods limit of detection for a reliable quantification.

## Discussion

In this study, we analyzed altered metabolite levels in blood samples of glioma patients collected years before diagnosed and compared that to healthy controls. We also examined how the metabolite levels change from years before diagnosis to the time of surgery. This approach enabled us to identify early metabolic markers that indicate glioma development years before diagnosis, and metabolites related to the progression of glioma up to the time of surgery.

For early metabolic markers of glioma development, we verify our previous findings of increased levels of metabolites related to energy turnover, years before glioma diagnosis^7^. These include elevated levels of lactate, pyruvate, fumarate and malate in the blood. Interestingly, fumarate was found to be significantly elevated in pre-diagnostic cases compared to controls, and their levels progressed to be significantly higher at surgery. This indicate that fumarate becomes even more pronounced as the disease progress. The same progression pattern with elevated levels towards surgery from already elevated levels years before diagnosis was also seen for pyruvate, lactate, urea and 2,3-dihydroxybutanoate. Methyl hexadecanoate was found to be significantly lower in pre-diagnostic glioma cases compared to controls, and significantly higher at the time of surgery. Methyl hexadecanoate is known to have anti-inflammatory properties^14^ and the higher levels at surgery could be due to a response to an inflammatory environment during glioma development. Previous metabolomics studies have indicated an imbalanced redox homeostasis in pre-diagnostic glioma cases^2,4,7^ with altered levels of metabolites of the glutathione metabolism^2^. Glutathione is an antioxidant and plays a key role in redox homeostasis^15^. In this study, we also found indications of an altered glutathione metabolism, with lower levels of pyroglutamate (5-oxoproline) years before diagnosis and elevated levels of methionine, serine, glycine seen at the time of surgery.

It is a well-known fact that the amino acid metabolism in gliomas is altered^16,17^. Here, we found elevated levels of amino acids and metabolites of amino acids metabolism at the time of surgery. Particularly BCKAs, ketoisoleucine and ketoleucine, that are metabolites from catabolism of the BCAAs, isoleucine and leucine^18^ by branched-chain amino acid aminotransferase, an enzyme that is expressed higher in glioblastoma tissue and higher expression levels are linked to poor survival^19^. Interestingly, the BCAA catabolism metabolite α-hydroxyisovalerate were significantly elevated pre-diagnostically and even higher levels were seen at of surgery for glioblastoma patients. Moreover, both α-hydroxyisovalerate and α-aminobutyrate are elevated in glioblastoma tissue^20^ and were here shown to be elevated in blood at surgery of glioblastoma patients. In addition to catabolic products of BCAAs, the levels of the amino acids asparagine, methionine, citrulline, serine, phenylalanine, glycine, proline, lysine, beta-alanine and pipecolinate/cycloleucine were elevated at the time of surgery. All these amino acids, except methionine and pipecolinate/cycloleucine, have been found to be higher in glioma tissue compared to brain tissue adjacent to tumor^20^. Higher levels of ketoleucine (2-oxoisocaproic acid) and α-hydroxyisovalerate (2-hydroxy-3-methylbutyric acid) in glioma tissue were also seen in the same study^20^. In general, we found elevated levels of metabolites of amino acid metabolism in blood at surgery that have been found elevated in glioma tissue. Amino acids can pass through the blood brain barrier through various mechanisms^21^ and can enter the blood stream and could affect the elevated levels we observe. However, the altered metabolite levels seen here in blood could be due to other causes, such as a rewired metabolism throughout the body as a consequence of the disease, and might not be directly applicable to earlier glioma detection and diagnosis. It should also be noted that most of the patients received the glucocorticoid betamethasone before surgery, which could alter blood metabolite levels. A previous study of a different glucocorticoid, dexamethasone, has shown to alter the levels of glucose, asparagine, proline, methionine, ketoleucine and phenylalanine in blood shortly after administrated^22^. The other 21 significantly metabolites we found altered at the time of surgery was not reported to be altered after administration of dexamethasone. Although the altered metabolism at surgery can depend on several factors, some of the altered metabolites seems to carry early diagnostic value as there altered levels years before diagnosis became even more pronounced at surgery (Fig. 4).

**Fig. 5:**
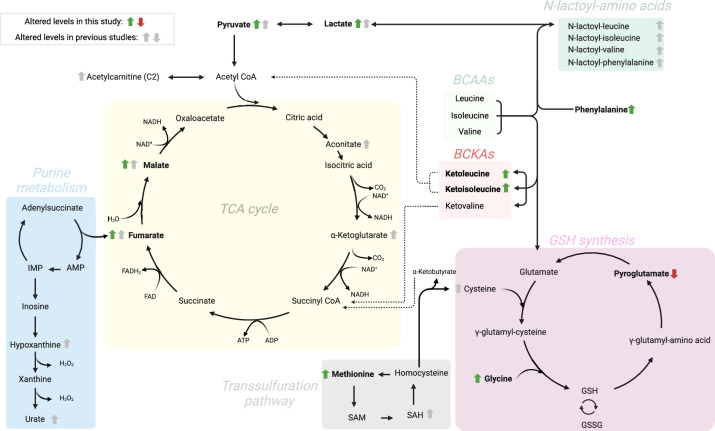
Schematic illustration of affected metabolic pathways during glioma development and progression**.** Altered metabolite levels found in this and earlier studies^2,7^. Altered metabolite levels occurs in closely related metabolic pathways that are linked to energy production (TCA cycle, purine metabolism, and lactate formation), redox balancing (GSH synthesis and transsulfuration pathway) and amino acid metabolism (BCAAs, BCKAs and N-lactoyl-amino acids). Green and red arrows depict elevated and decreased metabolite levels, respectively, found in this study whereas up/down pointing grey arrows indicate higher/lower levels found in previous studies. BCAAs = Branched chain amino acids. BCKAs = Branched chain α-keto acids. GSH = Glutathione. The illustration was created with BioRender.com.

As described, an altered energy state is seen in pre-diagnostic glioma cases and during the time of surgery. This altered energy state appears to be shift towards a catabolic state, as we see elevated levels of metabolites within the TCA cycle, protein- and amino acid catabolism, and increased lactate formation. In addition, we see indication of oxidative stress as metabolites within the glutathione metabolism are altered. As found here and in previous studies, the pre-diagnostic metabolic signature of glioma development seems to bear similarities to the metabolic signature of strenuous physical exercise^7–9^. Moreover, we see a large overlap of significant metabolites in our studies of pre-diagnostic glioma case and individuals diagnosed with mitochondrial disease, such as Mitochondrial encephalomyopathy lactic acidosis and stroke-like episodes^23^. Altered metabolites related to glioma development and progression found here and in previous studies^2,7^ together with altered metabolic pathways are mapped in Fig. 5.

Metabolite levels in blood are highly dynamic and depend on factors such as biological sex, age and fasting status. To adjust for these factors, we performed paired analysis with stringent matching of pre-diagnostic cases with controls based on biological sex, age and fasting status. Paired analysis was also performed for the surgery- and pre-diagnostic samples from the same individual, where the individual was fasting at both samplings. Even though biological sex difference is adjusted for by pairing to the same sex, the number of pre-diagnostic case–control pairs were higher for females (63.5%), which could influence the results. The opposite was true for the surgery and pre-diagnostic sample-pairs, with 59.3% males. However, the stringent matching approach applied here has previously shown to yield pre-diagnostic glioma metabolic markers that are independent on both biological sex and glioma subtype^7^.

This study confirms and validates previous findings in a new patient dataset. The main points of novelty are the identification of early metabolic markers that indicate glioma development, years before diagnosis, and metabolites related to the progression of glioma up to the time of surgery, in the same individuals. Specifically, we identify metabolites related to increased energy turnover, as highlighted by elevated levels of TCA-related metabolites in pre-diagnostic samples, and high levels of amino acids and metabolites of amino acid catabolism at glioma progression. These findings provide further insight into the metabolic alteration that occurs during glioma development and progression.

## Methods

### Study subjects and sample acquisition

EDTA-plasma samples from pre-diagnostic glioma cases and healthy controls were acquired from NSHDS^11^. NHSDS is a population-based and ongoing cohort in Västerbotten County, Sweden, with over 150 000 participants. Information about participant recruitment, study design, samples collection protocols and follow-up procedure has been published elsewhere^24^. We included pre-diagnostic glioma case samples collected up to eight years before diagnosis, together with matching controls. This resulted in 126 pre-diagnostic glioma case samples, from 105 individuals who had donated 1 or 2 blood samples, and 126 matched control samples. The study group consisted mainly of Caucasians individuals. Glioma cases were identified through cancer registries or via active follow up (ICD-7, topography: 193, histology: 475–476). Each case was paired randomly with a control that followed the set matching criteria. The control was alive and free of cancer at the time of diagnosis of the index case. Case–control matching was based on sex, age (± six months), BMI, time of sampling (± three months) and fasting status. Matched case–control pairs with opposite fasting status and suspected hemolyzed samples were not included.

We acquired EDTA-plasma samples collected on the day of surgery from the U-CAN biobank^12,25^. We selected surgery samples of individuals that also had pre-diagnostic samples in NSHDS, with a set limit of no more than 12 years before diagnosis. The U-CAN biobank was initiated later than NSHDS and therefore plasma samples were not collected at the time of surgery for all 126 individuals available. The latest pre-diagnostic sample was selected for individuals with more than one pre-diagnostic samples. Both the surgery and the pre-diagnostic sample were collected from fasting individuals. Suspected hemolyzed samples were removed from the analysis. This resulted in surgery samples (n = 40) and pre-diagnostic samples (n = 40) from 40 individuals, with pre-diagnostic samples collected up to 12 years before diagnosis with a median time of 4.4 years before diagnosis. Most of the patients received betamethasone before surgery. Additional drugs administered before surgery varied for each patient.

### Untargeted GC–MS metabolite analysis

For metabolite extraction, the plasma samples were divided into analytical batches, with matched pre-diagnostic case–control pairs and pre-diagnostic-surgery samples pairs kept within the same batch. Frozen 50 µl plasma samples in Eppendorf tubes were stored at -80 °C, and were placed on ice to thaw before extraction as previously described^2,20^. Extraction of metabolites was done by adding 450 µL cold methanol:water mixture (90:10 v/v, including internal standards (2.5–7.5 ng/µL)) and the tubes were shaken heavily at 30 Hz for 2 min using a bead mill (Retsch, MM 400). The samples were left on ice for 2 h before being centrifuged at 18,600 × g for 10 min at 4 °C. 100 µL of supernatant was collected from each Eppendorf tube and added to separate GC-vials. Supernatant was evaporated using a speedvac. The dry samples were derivatized by adding 15 µL methoxyamine in pyridine (15 µg/µL) and placed on a shaking machine for 10 min. The reaction was left for 16 h at room temperature. 15 µL MSTFA + 1% TMCS was then added and the vial was vortexed and left for 1 h at room temperature. 15 µL heptane with injection standard methyl stearate (15 ng/µL) was then added and the vial was vortexed. The samples were then analyzed using untargeted GC–MS.

For untargeted GC–MS analysis, we designed a constrained randomized run order^26^, where matched pre-diagnostic case–control pairs were analyzed directly adjacent to each other in a randomized order. The same procedure was applied for surgery samples and matched pre-diagnostic samples. The entire run order was randomized to achieve a balanced distribution of glioma subtype diagnosis, age, sex and time of sampling. To incorporate quality control measures, we included pooled quality control plasma samples, blank samples and serial dilutions throughout the analysis^27^. The samples from NSHDS and U-CAN were analyzed in the same analytical run.

The GC–MS analysis was performed as previously described^2,20^ using an Agilent 7890A gas chromatograph coupled to a Leco Pegasus HT time-of-flight mass spectrometer. 1 μL of sample was injected using a PAL autosampler in splitless mode at 260 °C, with a purge flow of 20 mL/min for 75 s. Helium carrier gas was kept at a flowrate of 1 mL/min. A 30 m DB-5MS column with 0.25 mm inner diameter and 0.25 μm film thickness was used for metabolite separation. The GC oven temperature program was set at 70 °C for 2 min, ramped at 20 °C/min until 320 °C, and then kept at 320 °C for 8 min. The temperature of the transfer line between the GC and the mass spectrometer was 250 °C. Electron impact ionization was performed at an energy level of 70 eV. The ion source temperature was 200 °C and detector voltage was set to 1820 V. Mass spectra from 50–800 m/z was recorded at 20 spectra/sec. An n-alkane series (C8-C40) was analyzed at the beginning, in the middle, and at the end of the analysis, and was used to calculate retention index (RI).

### Data processing and curation

Data processing was carried out as described previously^2,20^. Raw spectra were exported as NetCDF files and processed using in-house developed MATLAB scripts^28^. We used the area of the chromatographic peaks for quantification of relative amounts. The identities of the peaks were determined using mass spectral libraries from the Swedish Metabolomics Centre and the National Institute of Standards and Technology (NIST) (mainlib and replib libraries), using the NIST MS search 2.4 software. Identities were given to metabolites with spectral match score > 700, RI value within 25 units from the reference value, and all major ion fragments present in correct spectral intensities.

The data was curated before statistical analysis. Serial dilution of pooled quality control plasma samples was used to evaluate linearity of detection and to exclude molecular features not correlating with a linear quantification. To minimize the influence of instrument drift, raw peak area data was normalized by the analytical batches. That is, for each batch, each metabolite was divided by the batch median level of that metabolite. Metabolites with missing values were given an imputed value of half of the minimum intensity detected of that metabolite. We assessed the robustness of the analysis by examining the relative standard deviation (RSD) for the detected metabolites in the pooled plasma quality control samples. Metabolites that had missing values in more than 20% of samples were excluded from statistical analysis^29,30^. Detected drugs were also removed before statistical analysis. Median RSD was 27.9% and 86.8% of the molecular features had an RSD below 50%. After the data curation was completed, 134 identified metabolites and 22 unidentified molecular features remained.

### Analysis of chemical standards of N-lactoyl amino acids

We analyzed chemical standards of four commercially available N-lactoyl-amino acids: N-lactoyl-phenylalanine (Merck), N-lactoyl-leucine (Enamine), N-lactoyl-valine and N-lactoyl-tyrosine (BOC Sciences). All chemical standards were of analytical grade and were analyzed on the same GC–MS instrument, using the same settings and derivatization protocol as for the analytical samples described above. All standards were analyzed two times separately in concentrations of 20 ng/µL and 40 ng/µL for structural identification of mass spectrum and recording of RI. Dilution series for all four N-lactoyl-amino acid standards were analyzed with concentration range of 100 ng/µL – 0.2 ng/µL. Internal standards with final concentrations of 5–15 ng/µL were added to all concentrations of N-lactoyl-amino acids. The dilution series was used as a calibration curve for semi-quantification of N-lactoyl-amino acids in the analytical samples from the untargeted GC–MS analysis. Ratios of each N-lactoyl-amino acid and internal standard were calculated and quantified using the ratios from the calibration curve.

### Statistics

To discover metabolites that early indicate glioma development, we performed dependent multivariate modeling using OPLS-EP^26^ on an effect matrix with differences of metabolite levels of pre-diagnostic case–control pairs within eight years to diagnosis in NSHDS. For OPLS-EP modeling, the 134 metabolites and 22 unidentified molecular features were used as X variables. Response variable Y consisted of a vector with ones only. The X variables were scaled to unit variance without centering and the Y variable was not scaled.

To discover metabolites related to glioma progression, we performed OPLS-DA and OPLS-EP on surgery samples and matched pre-diagnostic samples. Curated batch normalized data was used for the OPLS-DA and the effect matrix of calculated metabolite level differences of each individual’s surgery sample and pre-diagnostic sample were used for OPLS-EP. Metabolites that were detected in both the surgery and pre-diagnostic samples were included in the modeling (134 metabolites and 22 unidentified molecular features). For OPLS-DA modeling, X variables were scaled to unit variance and centered.

For the OPLS model with time between surgery and pre-diagnostic sample as Y variable, we used the effect matrix with calculated differences of metabolite levels between the paired samples. The X variables were scaled to unit variance but not centered, whereas the Y variable was scaled to unit variance and centered.

Model significance was calculated using leave one out CV-ANOVA (two-sided)^31^ and metabolite significance was calculated using loadings w from the models (two-sided), which is equivalent to paired sample *t*-test for one component OPLS-EP models and independent *t*-test for one component OPLS-DA models. The selection of number of components included in each OPLS model was based on the lowest CV-ANOVA *P*-value. All statistical tests performed were two-sided and P < 0.05 was considered as a significant change.

### Study approval

All study participants provided written informed consent and all samples were pseudonymized. The study was conducted in accordance with the ethical standards of the Helsinki Declaration. The study project was approved by the ethical review board of Umeå University (Dnr 2017–295-31 M).

## Supplementary Information

Supplementary Information.

## Data Availability

Data can be shared upon reasonable request to the corresponding authors. Publicly sharing of data is not permitted according to written informed consent, and access to data will require ethical approval.
