# Supplementary material for: Blood based metabolic markers of glioma from pre-diagnosis to surgery
Source: Sci Rep. 2024 Sep 5;14:20680. doi: 10.1038/s41598-024-71375-6 (PMC11377417; doi:10.1038/s41598-024-71375-6)
Supplement: Supplementary file 1 — Supplementary Information. [file 41598_2024_71375_MOESM1_ESM.pdf]

Blood based metabolic markers of glioma from pre-diagnosis to surgery

Sebastian Löding[1*], Henrik Antti[1], Rickard L Sjöberg[2], Beatrice Melin[3], Benny Björkblom[1*].

[1] Department of Chemistry, Umeå University, SE-901 87 Umeå, Sweden.

[2] Department of Clinical Science, Neurosciences, Umeå University, SE-901 85 Umeå, Sweden.

[3] Department of Diagnostics and Intervention, Oncology, Umeå University, SE-901 87 Umeå, Sweden.

*Corresponding authors:

Sebastian Löding, Department of Chemistry, Umeå University, Linnaeus väg 10, SE-901 87 Umeå, Sweden. + 46907869715, sebastian.jonsson@umu.se.

Benny Björkblom, Department of Chemistry, Umeå University, Linnaeus väg 10, SE-901 87 Umeå, Sweden. +46907866230, benny.bjorkblom@umu.se.

Supplementary information

Supplementary Table S1. Significantly altered metabolites in blood of pre-diagnostic glioblastoma cases within eight years to diagnosis.

Metabolites	*P*-value	Mean difference (%)	HMDB ID
**Malate**	0.001	11	HMDB0000156
**Fumarate**	0.004	11	HMDB0000134
**α-Hydroxyisovalerate**	0.009	17	HMDB0000407
2,3-Dihydroxybutanoate	0.013	7	HMDB0245394
**Pyruvate**	0.013	14	HMDB0000243
α-Hydroxybutyrate	0.013	13	HMDB0341410
1,5-Anhydrosorbitol	0.027	9	HMDB0002712
**Lactate**	0.032	15	HMDB0000190
Oxalate	0.040	7	HMDB0002329
β-Hydroxybutyrate	0.041	21	HMDB0000011

P-values and mean percentage difference were calculated from case-control pairs within eight years to diagnosis (n=79). Significance levels were calculated from loadings w of the OPLS-EP model (two-sided), which is equivalent to paired samples *t*-test. Metabolites in bold were significant both for glioma and glioblastoma separately. Underlined metabolites were significant for glioblastoma only.

Supplementary Table S2. Metabolites with significantly higher or lower levels at glioblastoma surgery.

Metabolites	P-value	Mean difference (%)	HMDB ID
Higher at the time of surgery			
**Pipecolinate/Cycloleucine**	0.010	35	HMDB0000070/ HMDB0062225
**Ketoisoleucine**	0.012	58	
**2,3-Dihydroxybutanoate**	0.013	17	HMDB0245394
**Methyl hexadecanoate**	0.016	57	HMDB0061859
**Glucose**	0.021	18	HMDB0000122
α-aminobutyrate	0.023	25	HMDB0000452
**Asparagine**	0.024	68	HMDB0033780
**Xylitol/Arabitol/Ribitol 1**	0.025	32	
**Xylulose/Ribulose**	0.025	23	
**Methionine**	0.031	19	HMDB0000696
**Serine**	0.032	23	HMDB0000187
**Fumarate**	0.040	55	HMDB0000134
α-hydroxyisovalerate	0.040	11	HMDB0000407
Isomaltose	0.052	127	HMDB0002923
Erythronate	0.055	18	HMDB0000613
Xylitol/Arabitol/Ribitol 2	0.063	102	
Citrulline	0.065	21	HMDB0000904
Lysine	0.070	27	HMDB0000182
Urea	0.082	20	HMDB0000294
Phenylalanine	0.083	15	HMDB0000159
Ketoleucine	0.090	13	HMDB0000695
Glycine	0.113	15	HMDB0000123
Proline	0.115	22	HMDB0251528
beta-Alanine	0.130	12	HMDB0000056
Lower at the time of surgery			
**Hexitol (421 m/z, RI 1946)**	0.012	-63	
**Glycerol 3-phosphoate**	0.046	-37	HMDB0000126
**Glucoheptose**	0.049	-24	
Monosacharide (319 m/z, RI 2103)	0.055	-12	
Unknown (331 m/z, RI 1971)	0.108	-21	

P-values and mean percentage difference were calculated from paired surgery samples (n=21) and pre-diagnostic samples (n=21) of glioblastoma patients within seven years to diagnosis. Significance levels were calculated from loadings w of the model (two-sided), which is equivalent to paired samples t-test. Metabolites in bold were significant for all glioma and glioblastoma separately. Underlined metabolites were significant for glioblastoma only. Non-bold non-underlines metabolites were significant for all glioma, but not for glioblastoma separately.

a

Model	Y variable	Surgery samples	Pre-dx samples	Time to dx limit	Median time to dx	Components (Pred + Orth)	R_2Y	Q^2	*P*-value (CV-ANOVA)
OPLS	Time between paired samples	n = 27	n = 27	7 years	3.2 years	1+0	0.436	0.14	0.17

b

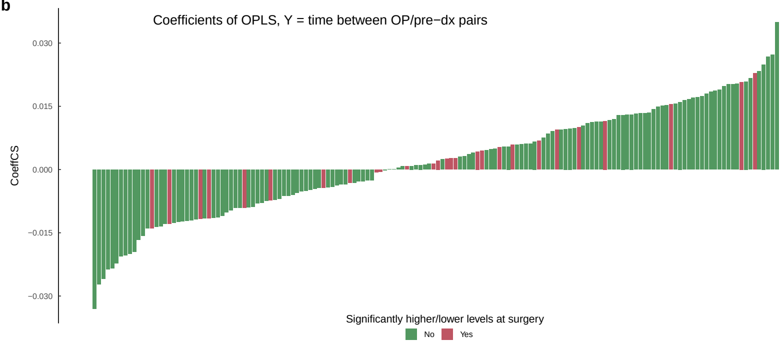

c

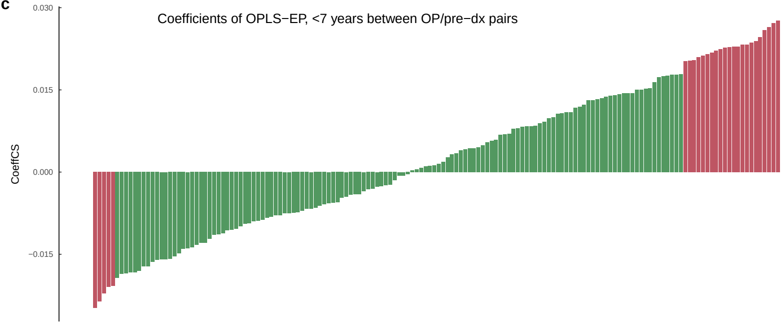

Supplementary Figure S1. Metabolite levels related to time between samples. (**a**) OPLS time model characteristics. The matrix of calculated metabolite levels of surgery and pre-diagnostic samples was used to generate an OPLS model with time between sample pairs as Y variable. (**b**) Coefficient of the OPLS time model. A larger positive or negative coefficient value means that a metabolite has a larger positive or negative correlation with time, respectively. Metabolites that were significantly higher or lower at the time of surgery compared to years before diagnosis are colored in red, and were in general not correlated with time. (**c**) Coefficients of the OPLS-EP model with surgery and pre-diagnostic sample pairs with the 7 years limit between samples. Metabolites with large coefficient values are correlated with disease progression. OP = surgery. pre-dx = pre-diagnostic.

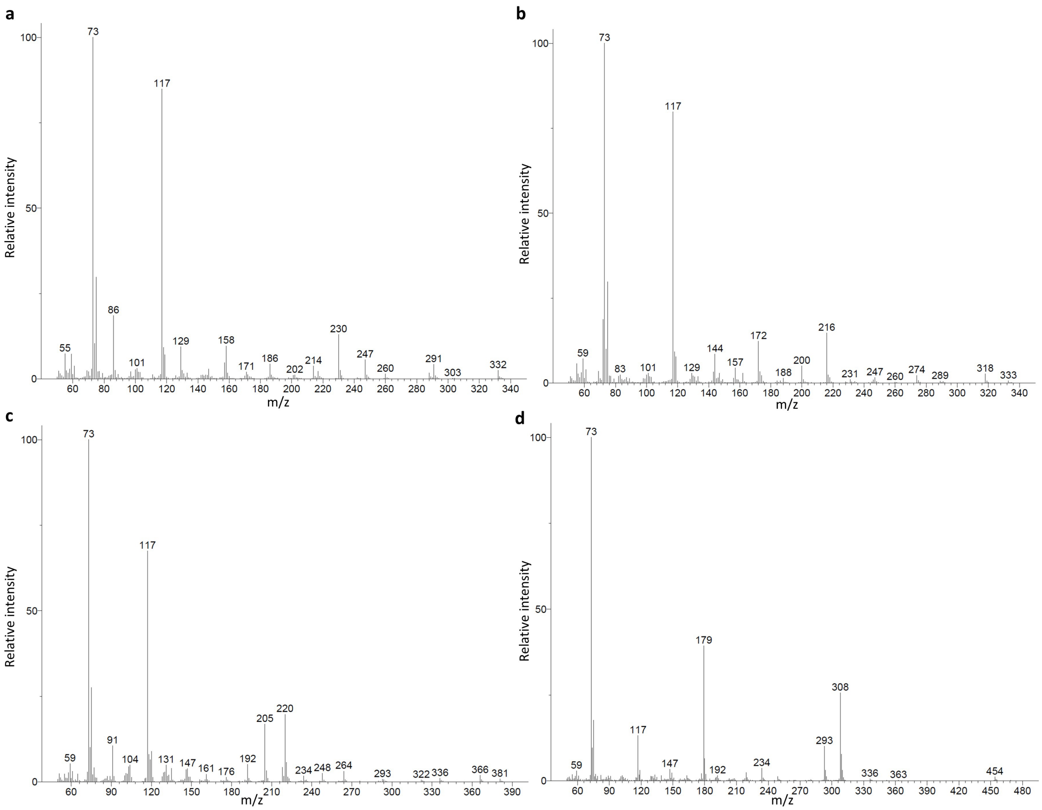

Supplementary Figure S2. Recorded mass spectra of N-lactoyl-amino acids. Mass spectra of (**a**) N-lactoyl-leucine, (**b**) N-lactoyl-valine, (**c**) N-lactoyl-phenylalanine, and (**d**) N-lactoyl-tyrosine. All N-lactoyl-amino acids were derivatized using methoxyamine in pyridine and MSTFA with 1% TMCS (see Methods for details).
